# Osteosarcopenia as a Risk Factor for Atrial Fibrillation: A Multi-Cohort Study

**DOI:** 10.1016/j.jacasi.2026.08.006

**Published:** 2026-09-16

**Authors:** Kyung-Yeon Lee, Tae-Min Rhee, JungMin Choi, Hyo-Jeong Ahn, So-Ryoung Lee, Heesun Lee, Su-Yeon Choi, Seil Oh, Gregory Y.H. Lip, Eue-Keun Choi

**Affiliations:** a Department of Internal Medicine, Korea University Guro Hospital, Seoul, Republic of Korea; b Department of Internal Medicine, Seoul National University College of Medicine, Seoul, Republic of Korea; c Department of Internal Medicine, Seoul National University Hospital Healthcare System Gangnam Center, Seoul, Republic of Korea; d Department of Internal Medicine, Seoul National University Hospital, Seoul, Republic of Korea; e Liverpool Centre for Cardiovascular Science at University of Liverpool, Liverpool John Moores University and Liverpool Chest and Heart Hospital, Liverpool, United Kingdom; f Department of Clinical Medicine, Aalborg University, Aalborg, Denmark

**Keywords:** arrhythmia, atrial fibrillation, cardiovascular disease, osteoporosis, osteosarcopenia, sarcopenia

## Abstract

**Background:**

Osteosarcopenia, characterized by concurrent reductions in skeletal muscle mass and bone mineral density, is an emerging geriatric syndrome. Although sarcopenia and osteoporosis have been individually linked to atrial fibrillation (AF), the association between osteosarcopenia and incident AF remains unclear.

**Objectives:**

The authors aimed to investigate the association between osteosarcopenia and incident AF and to evaluate its incremental prognostic value beyond established AF risk factors.

**Methods:**

In this prospective cohort study using the UK Biobank, participants without previous AF were included at baseline. A replication analysis was conducted in the H-PEACE (Health and Prevention Enhancement) cohort. Participants were categorized into 4 groups: reference, sarcopenia alone, osteoporosis alone, and osteosarcopenia. The primary endpoint was incident AF during a median follow-up of 13.8 [13.1;14.6] years. Multivariable Cox regression and risk prediction analyses were performed.

**Results:**

Among 482,137 participants (mean age 56.4 ± 8.1 years; 45.2% male), osteosarcopenia was present in 0.65%. In the fully adjusted 4-group model, osteoporosis (adjusted HR [aHR]: 1.18; 95% CI: 1.03-1.36) and, most strongly, osteosarcopenia (aHR 1.33; 95% CI 1.09-1.62) were independently associated with incident AF, whereas sarcopenia alone was not (aHR: 1.05; 95% CI: 0.99-1.10). These findings were consistent in the H-PEACE cohort (adjusted OR: 2.10; 95% CI: 1.06-4.13; *P* = 0.032).

**Conclusions:**

Osteosarcopenia was independently associated with incident AF and provided incremental prognostic information beyond traditional risk factors, although the improvement in discrimination was limited. These findings support tailored AF screening in patients with osteosarcopenia to facilitate earlier diagnosis and treatment.

Osteosarcopenia is a condition characterized by both low muscle quality and quantity (sarcopenia), as well as low bone density (osteoporosis). The prevalence of osteosarcopenia ranges from 5% to 37% in community-dwelling older adults, but it is still considered underdiagnosed.^1^ Osteosarcopenia is expected to increase as an age-related syndrome, contributing to a rising global health burden. Its importance in the clinical field is increasing because of its relationship to disability, frailty, hospitalization, and death.^2^, ^3^, ^4^

Sarcopenia and osteoporosis are related to cardiovascular disease (CVD) and share risk factors in terms of metabolic syndrome, such as age, smoking, obesity, physical inactivity, hypertension, diabetes mellitus, and dyslipidemia.^5^^,^^6^ Given this background, it can be assumed that osteosarcopenia is also associated with CVD, as it is a combined condition of sarcopenia and osteoporosis. However, there is a paucity of studies evaluating the correlation between osteosarcopenia and CVD. One previous study demonstrated that osteosarcopenia was independently associated with coronary artery disease^7^ and another showed a 3-fold increase in 1-year mortality in patients with osteosarcopenia who underwent transcatheter aortic valve replacement.^8^

Atrial fibrillation (AF) is one of the CVDs associated with adverse clinical outcomes such as heart failure, myocardial infarction, stroke, and death.^9^^,^^10^ The prevalence of AF increases with age, and because AF and osteosarcopenia share several risk factors, there may be a potential coexistence and interrelation between the 2 conditions.^11^^,^^12^ However, the association between osteosarcopenia and AF has yet to be evaluated. Thus, we aimed to evaluate the impact of osteosarcopenia on the risk of incident AF.

## Methods

### Study population

This study used the UK Biobank, which includes more than 500,000 participants, aged 40 to 69 years, registered with the UK National Health Service and living within 25 miles of an assessment center between 2006 and 2010. The detailed study protocols for the UK Biobank have been described in previous publications.^13^ Of the initial 502,386 participants, individuals with a previous diagnosis of AF or missing data related to muscle quality (hand grip strength) and quantity (appendicular lean mass [ALM]) were excluded. The final study population consisted of 482,137 participants who were divided into 4 groups based on the presence of sarcopenia and osteoporosis: 1) reference group without sarcopenia or osteoporosis; 2) sarcopenia group with sarcopenia alone; 3) osteoporosis group with osteoporosis alone; and 4) osteosarcopenia group with both sarcopenia and osteoporosis (Figure 1).

**Figure 1:**
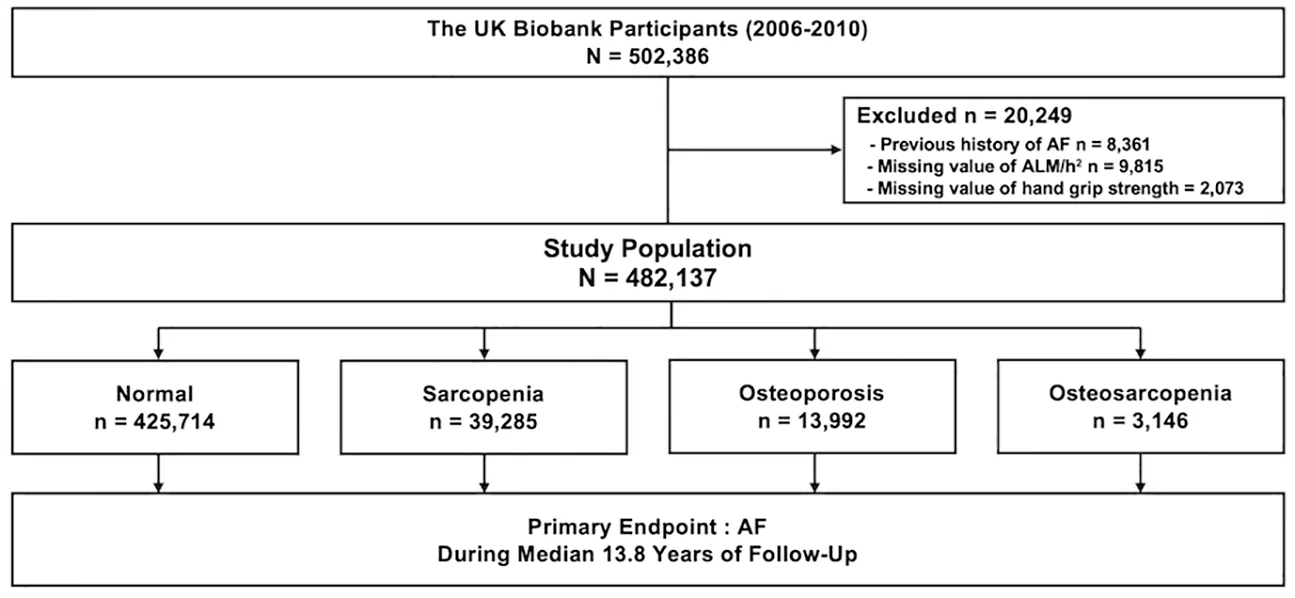
Study Flow Diagram The flow diagram of the present study is shown. AF = atrial fibrillation; ALM = appendicular lean mass.

Ethical approval for this research was obtained from the Northwest Multi-Center Research Ethics Committee and sanctioned by the UK Biobank's review committee (application number 76593). The study was also approved by the National Health Service National Research Ethics Service on June 17, 2011 (Ref 11/NW/0382), with an extension granted on May 10, 2016 (Ref 16/NW/0274). All participants provided informed consent.

Data from the H-PEACE (Health and Prevention Enhancement) cohort of the Seoul National University Hospital Healthcare System Gangnam Center were used for the replication analysis. The H-PEACE cohort is a retrospective, population-based study that collected large-scale data from health examinees in South Korea.^14^ Comprehensive information was obtained through structured questionnaires, physical examinations, laboratory tests, and imaging studies. Among the 178,266 eligible participants enrolled between 2003 and 2023, those who underwent both dual-energy X-ray absorptiometry and body composition analysis using bioelectrical impedance analysis (BIA) on the same day of their health checkup were included in the analysis. After excluding individuals with a previous diagnosis of AF, 32,961 participants comprised the cohort for replication analysis. Similar to the UK Biobank analysis, participants were categorized into 4 groups: reference, sarcopenia alone, osteoporosis alone, and osteosarcopenia. The study was approved by the Institutional Review Board of Seoul National University Hospital and the Seoul National University Hospital Healthcare System Gangnam Center (IRB Nos. 1605-115-763 and H-2410-130-1578).

### Definition of osteosarcopenia

Sarcopenia was defined according to the EWGSOP2 (European Working Group on Sarcopenia in Older People 2) criteria.^15^ Low muscle strength was assessed using the mean hand grip strength, with cutoff values set at less than 27 kg for men and less than 16 kg for women. Low muscle quantity was measured by calculating the ALM divided by height squared, with cutoff values of less than 7 kg/m^2^ for men and less than 5.5 kg/m^2^ for women. ALM was calculated using an equation that estimates ALM based on appendicular fat-free mass: ALM (kg) = (0.958 × [appendicular fat-free mass (kg)]) − (0.166 × G) − 0.308, where G is 0 for female and 1 for male individuals.^16^ Sarcopenia was confirmed when participants had both low muscle strength and low muscle quantity. Osteoporosis was defined using International Classification of Diseases, 10^th^ Revision (ICD-10) diagnostic codes from linked in-hospital and outpatient clinic records before baseline, because systematic dual-energy X-ray absorptiometry–based bone mineral density measurements were not available for the entire cohort. Osteosarcopenia was identified in participants who met the criteria for both sarcopenia and osteoporosis.

For the replication analysis, because the H-PEACE cohort did not provide muscle strength information, we only used muscle quantity data from the BIA to define sarcopenia. We calculated ALM/height2 from BIA results and used the same cutoff values (<7 kg/m^2^ for men and <5.5 kg/m^2^ for women). Osteoporosis and osteosarcopenia were defined using the same criteria as the UK Biobank analysis.

### Definitions of covariates and outcomes

Supplemental Table 1 provides detailed information on the covariates and outcomes. The primary outcome was the incidence of AF, identified using the ICD-10 code I48, as recorded in the UK Biobank registry data during the follow-up period. Over a median follow-up period of 13.8 years (IQR: 13.1-14.6 years), participants were censored at the earliest occurrence of AF, the end of the follow-up period, or death. In the replication analysis, because the H-PEACE cohort did not provide the exact date of AF onset but instead collected data retrospectively during each health checkup session, precise time-to-event information was not available and time-to-event analyses were not performed. Therefore, we conducted a cross-sectional analysis using incident AF cases identified between the time of enrollment and the end of follow-up (January 2025). The median follow-up duration was 7.2 years (IQR: 4.2-10.8 years).

### Statistical analyses

Continuous variables were reported as mean ± SD or median (IQR) and categorical variables were presented as counts and percentages. Categorical variables were analyzed using the chi-square test, and 1-way analysis of variance was applied to compare the 4 groups for continuous variables. The Kaplan-Meier method estimated the cumulative incidence of AF, and differences between groups were assessed using the log-rank test. Multivariable Cox proportional hazards models were used to estimate HRs and 95% CIs for incident AF associated with osteosarcopenia. The proportional hazards assumption was evaluated using Schoenfeld residuals. Although the global test indicated some deviation from proportionality, the main exposure variable (osteosarcopenia) satisfied the proportional hazards assumption. To assess potential nonlinear associations, natural cubic spline functions were applied using the ns() function in R with 3 degrees of freedom for handgrip strength and ALM indexed by height squared (ALM/height^2^). Univariate and multivariate binary logistic regression analyses identified significant factors associated with the incidence of AF. To evaluate the incremental predictive value of osteosarcopenia beyond the CHARGE-AF score, 2 prediction models were compared. Model 1 included the CHARGE-AF (Cohorts for Heart and Aging Research in Genomic Epidemiology Atrial Fibrillation) score alone, whereas model 2 additionally included body composition status as a 4-category variable (reference, sarcopenia, osteoporosis, and osteosarcopenia). Model discrimination was assessed using the area under the receiver operating characteristic curve (AUROC) and Harrell's C-index, and reclassification was evaluated using the category-free net reclassification improvement (NRI). Differences in AUROCs between prediction models were assessed using DeLong's test. Adjustments were made for various covariates, including age, sex, body mass index (BMI), waist circumference, hip circumference, self-reported walking speed, falls in the past year, physical activity, smoking status, alcohol consumption, Charlson Comorbidity Index score, ethnicity, history of hypertension, diabetes mellitus, dyslipidemia, myocardial infarction, heart failure, stroke, levels of hemoglobin, glucose, HbA1c, cholesterol, high-density lipoprotein cholesterol, low-density lipoprotein cholesterol, triglycerides, creatinine, albumin, vitamin D, and osteoporosis medication. Because the replication cohort was based on retrospectively collected health examination data with limited information on follow-up duration and timing of AF onset, time-to-event analysis was not feasible. The replication analysis, therefore, included both univariable and multivariable logistic regression analyses to estimate the ORs and 95% CIs for AF according to sarcopenia, osteoporosis, and osteosarcopenia. The multivariable logistic regression model was adjusted for age, sex, BMI, waist circumference, smoking status, alcohol consumption, systolic and diastolic blood pressures, and levels of hemoglobin, glucose, HbA1c, total cholesterol, triglycerides, creatinine, albumin, and use of osteoporosis medication. The AUROC and the category-free NRI were used to evaluate the incremental discriminative ability of osteosarcopenia in predicting AF in the replication cohort. Prespecified subgroup analyses were performed according to clinically relevant variables, including age, sex, and key comorbidities, to evaluate the consistency of the model performance across different patient groups. These subgroups were selected based on their known associations with the outcome and potential effect modification. To formally assess effect modification, multiplicative interaction terms between the main predictor and subgroup variables were included in the regression models, and statistical significance of interactions was evaluated accordingly. A *P* value of less than 0.05 was considered statistically significant. All statistical analyses were performed using R version 4.4.3.

## Results

Baseline characteristics of the 4 groups are presented in Table 1. Among the 482,137 participants, the mean age was 56.4 ± 8.1 years, and 45.2% (217,784 of 482,137) were male. The prevalence of sarcopenia alone in our study was 8.1% (39,285 of 482,137), osteoporosis alone 2.9% (13,992 of 482,137), and osteosarcopenia 0.65% (3,146 of 482,137). Compared with the reference group, participants in the osteosarcopenia group were older, had lower waist and hip circumferences, reduced hand grip strength, slower walking speed, and a higher frequency of falls in the past year. In addition, the osteosarcopenia group had a higher prevalence of comorbidities, including hypertension, diabetes mellitus, dyslipidemia, and a history of myocardial infarction, heart failure, and stroke. There was no significant difference in smoking status between the reference and osteosarcopenia groups; however, the osteosarcopenia group reported lower alcohol consumption.

**Table 1:** Baseline Characteristics

	Total (N = 482,137)	Reference (n = 425,714)	Sarcopenia (n = 39,285)	Osteoporosis (n = 13,992)	Osteosarcopenia (n = 3,146)	*P* Value
Age, y	56.4 ± 8.1	56.0 ± 8.1	59.5 ± 7.3	60.4 ± 6.6	62.1 ± 5.9	<0.001
Male	217,784 (45.2)	200,486 (47.1)	14,578 (37.1)	2,284 (16.3)	436 (13.9)	<0.001
BMI, kg/m^2^						<0.001
<18.5	2,471 (0.5)	1872 (0.4)	299 (0.8)	222 (1.6)	78 (2.5)	
18.5 ≤BMI <25	157,827 (32.7)	138,981 (32.6)	11,292 (28.7)	6,367 (45.5)	1,187 (37.7)	
25 ≤BMI <30	205,082 (42.5)	183,080 (43.0)	15,788 (40.2)	5,049 (36.1)	1,165 (37.0)	
≥30	116,757 (24.2)	101,781 (23.9)	11,906 (30.3)	2,354 (16.8)	716 (22.8)	
Waist circumference, cm	90.1 ± 13.4	90.2 ± 13.3	91.7 ± 14.1	84.6 ± 12.9	86.7 ± 13.5	<0.001
Hip circumference, cm	103.3 ± 9.2	103.4 ± 9.0	104.0 ± 10.5	101.1 ± 9.3	101.6 ± 10.1	<0.001
Hand grip strength, kg	30.6 ± 11.0	32.3 ± 10.3	15.9 ± 5.9	25.5 ± 7.6	12.9 ± 4.9	<0.001
Self-reported walk speed						<0.001
Slow pace	36,813 (7.7)	25,971 (6.1)	8,374 (21.8)	1,437 (10.4)	1,031 (33.9)	
Steady average pace	253,224 (52.9)	223,559 (52.8)	21,049 (54.8)	7,185 (51.8)	1,431 (47.1)	
Brisk pace	188,756 (39.4)	173,955 (41.1)	8,978 (23.4)	5,245 (37.8)	578 (19.0)	
Falls in past year						<0.001
0	386,777 (80.6)	347,794 (82.0)	26,874 (69.2)	10,237 (73.5)	1872 (60.2)	
1	62,786 (13.1)	53,206 (12.5)	6,533 (16.8)	2,434 (17.5)	613 (19.7)	
2+	30,523 (6.4)	23,225 (5.5)	5,409 (13.9)	1,264 (9.1)	625 (20.1)	
Regular physical activity	210,336 (54.4)	189,854 (55.0)	13,558 (47.2)	5,940 (54.9)	984 (42.9)	<0.001
Current smoker	50,837 (10.5)	44,995 (10.6)	4,333 (11.0)	1,191 (8.5)	318 (10.1)	<0.001
Daily drinking	97,715 (20.3)	88,560 (20.8)	6,141 (15.6)	2,597 (18.6)	417 (13.3)	<0.001
CCI score	1.4 ± 1.2	1.4 ± 1.2	1.9 ± 1.4	2.1 ± 1.6	2.5 ± 1.6	<0.001
Ethnicity						<0.001
White	427,728 (88.8)	379,404 (89.2)	33,223 (84.8)	12,424 (88.9)	2,677 (85.3)	
Asian	19,634 (4.1)	17,226 (4.0)	1,662 (4.2)	614 (4.4)	132 (4.2)	
Black	14,759 (3.1)	12,543 (2.9)	1,579 (4.0)	504 (3.6)	133 (4.2)	
Mixed	13,067 (2.7)	11,430 (2.7)	1,297 (3.3)	270 (1.9)	70 (2.2)	
Others	6,452 (1.3)	4,740 (1.1)	1,421 (3.6)	166 (1.2)	125 (4.0)	
Townsend deprivation index [IQR]	−2.2 [−3.7;0.5]	−2.2 [−3.7;0.4]	−1.4 [−3.3;1.8]	−2.2 [−3.7;0.5]	−1.6 [−3.2;1.4]	<0.001
Household income before tax, pound						<0.001
<18,000	91,703 (22.4)	75,080 (20.6)	12,084 (39.5)	3,444 (30.9)	1,095 (46.6)	
18,000-30,999	103,835 (25.4)	91,473 (25.1)	8,337 (27.3)	3,328 (29.8)	697 (29.7)	
31,000-51,999	107,262 (26.2)	98,437 (27.0)	6,018 (19.7)	2,475 (22.2)	332 (14.1)	
52,000-100,000	84,023 (20.5)	78,975 (21.6)	3,363 (11.0)	1,506 (13.5)	179 (7.6)	
>100,000	22,264 (5.4)	21,056 (5.8)	755 (2.5)	408 (3.7)	45 (1.9)	
Hypertension	140,616 (29.2)	120,033 (28.2)	15,256 (38.8)	4,086 (29.2)	1,241 (39.4)	<0.001
Diabetes mellitus	25,478 (5.3)	20,502 (4.8)	4,128 (10.5)	581 (4.2)	267 (8.5)	<0.001
Dyslipidemia	92,177 (19.1)	77,057 (18.1)	11,212 (28.5)	2,952 (21.1)	956 (30.4)	<0.001
Previous history of MI	10,306 (2.1)	8,492 (2.0)	1,432 (3.6)	273 (2.0)	109 (3.5)	<0.001
Previous history of HF	1,565 (0.3)	1,185 (0.3)	299 (0.8)	55 (0.4)	26 (0.8)	<0.001
Previous history of stroke	7,739 (1.6)	5,890 (1.4)	1,407 (3.6)	293 (2.1)	149 (4.7)	<0.001
Hb	14.2 ± 1.2	14.2 ± 1.2	13.9 ± 1.2	13.7 ± 1.1	13.5 ± 1.2	<0.001
Fasting glucose	5.1 ± 1.2	5.1 ± 1.2	5.3 ± 1.6	5.1 ± 1.1	5.2 ± 1.3	<0.001
HbA1c	36.1 ± 6.7	35.9 ± 6.5	37.8 ± 8.3	36.2 ± 5.7	37.5 ± 7.1	<0.001
Total cholesterol	5.7 ± 1.1	5.7 ± 1.1	5.6 ± 1.2	5.9 ± 1.1	5.7 ± 1.2	<0.001
HDL	1.5 ± 0.4	1.4 ± 0.4	1.4 ± 0.4	1.6 ± 0.4	1.6 ± 0.4	<0.001
LDL	3.6 ± 0.9	3.6 ± 0.9	3.5 ± 0.9	3.6 ± 0.9	3.5 ± 0.9	<0.001
TG	1.7 ± 1.0	1.7 ± 1.0	1.8 ± 1.1	1.6 ± 0.9	1.7 ± 0.9	<0.001
Creatinine	72.2 ± 18.0	72.5 ± 16.7	70.6 ± 26.3	67.6 ± 23.2	67.2 ± 26.2	<0.001
Albumin	45.2 ± 2.6	45.3 ± 2.6	44.7 ± 2.7	44.9 ± 2.7	44.2 ± 2.9	<0.001
Vitamin D	48.7 ± 21.1	48.6 ± 20.9	45.9 ± 21.0	59.0 ± 22.0	56.8 ± 22.8	<0.001
Osteoporosis medication	12,208 (2.5)	0 (0.0)	0 (0.0)	10,013 (71.6)	2,195 (69.8)	<0.001
FU duration, y						
Mean ± SD	13.5 ± 2.0	13.5 ± 2.0	13.1 ± 2.4	13.3 ± 2.4	12.7 ± 2.9	<0.001
Median [IQR]	13.8 [13.1;14.6]	13.9 [13.1;14.6]	13.6 [12.8;14.3]	13.8 [13.0;14.6]	13.5 [12.7;14.3]	<0.001

Data summarized as mean ± SD or n (%) unless otherwise indicated.

BMI = body mass index; CCI = Charlson comorbidity index; FU = follow-up; Hb = hemoglobin; HDL = high-density lipoprotein; HF = heart failure; LDL = low-density lipoprotein; MI = myocardial infarction; TG = triglycerides.

### Association of sarcopenia and osteoporosis and incident AF

Supplemental Table 2 presents the association between sarcopenia, osteoporosis, and the incidence of AF. The incidence rate (IR) was higher in participants with sarcopenia compared with those without, with an IR of 6.60 per 1,000 person-years (PY) in the sarcopenic group vs 4.30 per 1,000 PY in the nonsarcopenic group. Sarcopenia was significantly associated with an increased incidence of AF, with an adjusted HR (aHR) of 1.05 (95% CI: 1.00-1.11; *P* = 0.015). The IR for osteoporosis was 5.95 per 1,000 PY, and osteoporosis was also significantly associated with incident AF, with an aHR of 1.20 (95% CI: 1.05-1.37; *P* = 0.008).

### Association of osteosarcopenia and incident AF

Table 2 presents the association between osteosarcopenia and the incidence of AF. The IRs of AF were highest in the osteosarcopenia group, followed by the sarcopenia and osteoporosis groups, with rates of 7.87, 6.50, and 5.55 per 1,000 PY, respectively.

**Table 2:** Incidence and Risk of Atrial Fibrillation According to Groups Divided by Sarcopenia and Osteoporosis Status

	n	Event	IR, %IR per 1,000 PY (95% CI)	Unadjusted HR		Adjusted HR (Model 1)		Adjusted HR (Model 2)	
				HR (95% CI)	*P* Value	HR (95% CI)	*P* Value	HR (95% CI)	*P* Value
Reference	425,714	23,983	4.26 (4.21-4.32)	1 (Reference)		1 (Reference)		1 (Reference)	
Sarcopenia	39,285	3,227	6.50 (6.28-6.73)	1.52 (1.46-1.57)	<0.001	1.23 (1.18-1.27)	<0.001	1.05 (0.99-1.10)	0.077
Osteoporosis	13,992	1,000	5.55 (5.21-5.90)	1.30 (1.22-1.39)	<0.001	1.17 (1.09-1.24)	<0.001	1.18 (1.03-1.36)	0.020
Osteosarcopenia	3,146	302	7.87 (7.00-8.81)	1.84 (1.64-2.06)	<0.001	1.49 (1.33-1.67)	<0.001	1.33 (1.09-1.62)	0.005

Adjusted HRs were calculated using multivariable Cox regression models

Model 1: Adjusted for age and sex.

Model 2: Adjusted for age, sex, body mass index, waist circumference, hip circumference, self-reported walking speed, falls in the past year, physical activity, smoking status, alcohol consumption, Charlson comorbidity index score, ethnicity, history of hypertension, diabetes mellitus, dyslipidemia, myocardial infarction, heart failure, stroke, levels of hemoglobin, glucose, HbA1c, cholesterol, high-density lipoprotein, low-density lipoprotein, triglycerides, creatinine, albumin, vitamin D, and osteoporosis medication.

IR = incidence rate; PY = person-year.

The risk of incident AF was significantly higher in the osteosarcopenia group, with an aHR of 1.33 (95% CI: 1.09-1.62; *P* = 0.005) compared with the reference group, followed by the osteoporosis group (aHR: 1.18; 95% CI: 1.03-1.36; *P* = 0.020). Sarcopenia was not significantly associated with incident AF (aHR: 1.05; 95% CI: 0.99-1.10; *P* = 0.077). The adjusted Kaplan-Meier curves demonstrate that sarcopenia, osteoporosis, and osteosarcopenia were all significantly associated with the incidence of AF. Osteosarcopenia was associated with the highest risk of incident AF, followed by sarcopenia and osteoporosis (log-rank *P* <0.001) (Figure 2).

**Figure 2:**
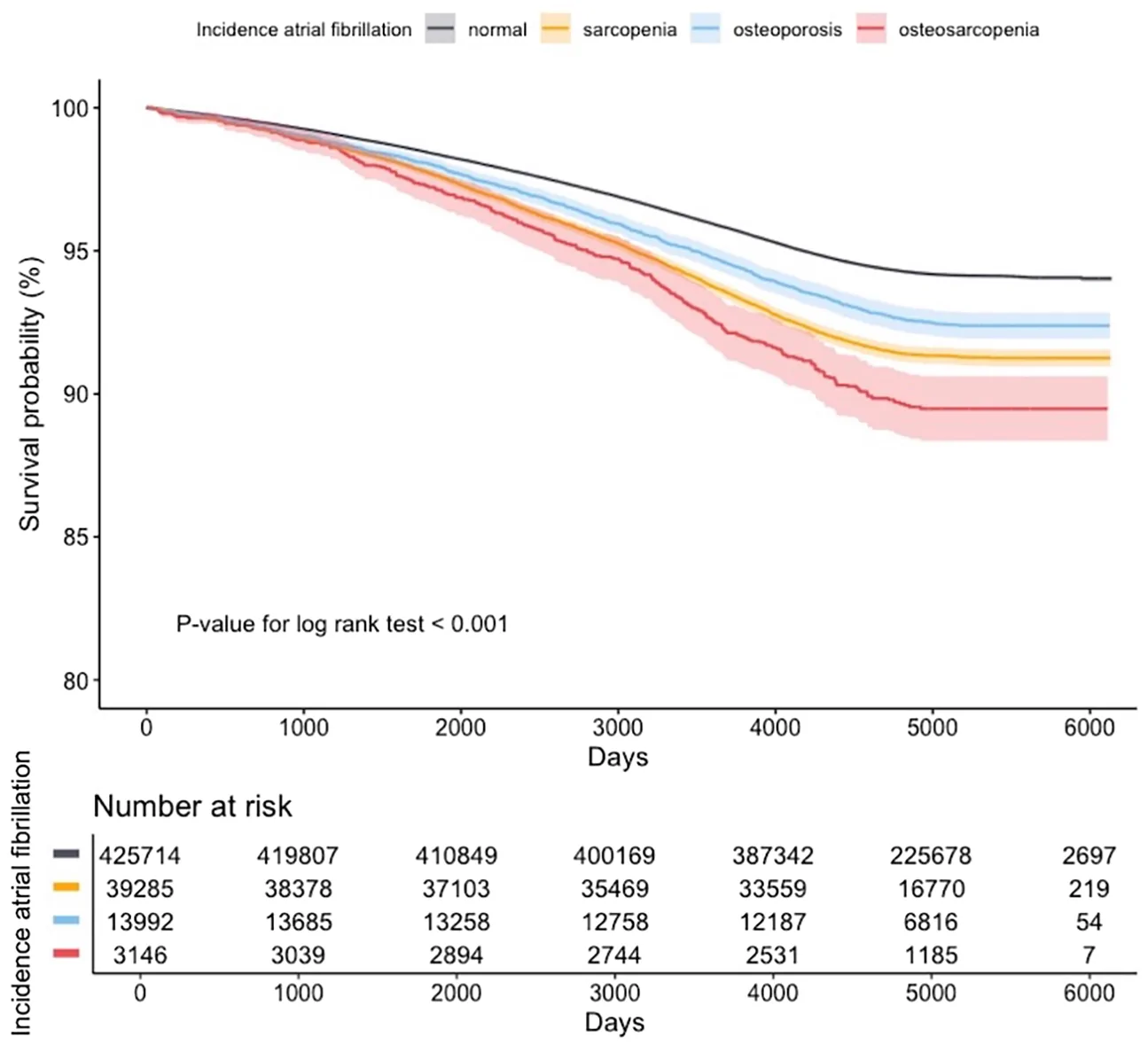
Adjusted Risk of Incident Atrial Fibrillation Kaplan-Meier curves illustrate the cumulative incidence of atrial fibrillation according to body composition status (reference, sarcopenia, osteoporosis, and osteosarcopenia) in the UK Biobank cohort. Curves were adjusted using multivariable Cox proportional hazards models. Participants with osteosarcopenia showed the highest cumulative incidence of atrial fibrillation during follow-up, whereas the reference group had the lowest incidence, supporting the independent association between osteosarcopenia and incident atrial fibrillation.

### Replication analysis in the H-PEACE cohort

Baseline characteristics in the replication cohort are summarized in Supplemental Table 3. Among 32,961 participants (mean age 54.4 ± 10.7 years; 27.9% [9,211 of 32,961] male), the prevalence of sarcopenia alone was 11.3% (3,734 of 32,961), osteoporosis alone 7.1% (2,342 of 32,961), and osteosarcopenia 2.1% (680 of 32,961). Compared with the reference group, individuals with osteosarcopenia were significantly older, had lower BMI, and smaller waist circumference. The osteosarcopenia group also had lower rates of current smoking and alcohol consumption. Notably, comorbid conditions such as hypertension and dyslipidemia were more prevalent in this group, whereas diabetes mellitus prevalence was similar across groups.

In the replication analysis (Supplemental Table 4), AF rates were 1.97% (87 of 4,414) for sarcopenia and 2.45% (74 of 3,022) for osteoporosis. Sarcopenia was not significantly associated with incident AF (adjusted OR: 1.05; 95% CI: 0.76-1.46; *P* = 0.771), and osteoporosis showed an association of borderline significance (adjusted OR: 1.70; 95% CI: 0.99-2.92; *P* = 0.055).

In the replication analysis (Supplemental Table 5), those with osteosarcopenia had the highest AF incidence (3.09% [21 of 680]), followed by osteoporosis (2.26% [53 of 2,342]), reference (2.03% [532 of 26,205]), and sarcopenia alone (1.77% [66 of 3,734]). Osteosarcopenia was significantly associated with increased AF risk in multivariable analysis (adjusted OR: 2.10; 95% CI: 1.06-4.13; *P* = 0.032), whereas sarcopenia and osteoporosis alone were not.

### The risk factors for incident AF

Supplemental Table 6 presents the risk factors for incident AF in the study cohort. In the multivariable analysis, sarcopenia, osteoporosis, osteosarcopenia, age, male sex, obesity, hypertension, diabetes mellitus, dyslipidemia, previous history of heart failure, daily alcohol consumption, and current smoking were independently associated with incident AF.

### Additive predictive value of osteosarcopenia on AF prediction

Table 3 presents an analysis of the additive predictive value of osteosarcopenia on the risk of incident AF. Model 1 used the CHARGE-AF score as a baseline. Model 2 added osteosarcopenia to model 1. The AUROC was essentially unchanged (0.758 [0.755-0.760] and 0.758 [0.755-0.761] for models 1 and 2, respectively), and Harrell's C-index was likewise nearly identical (0.756 [0.754-0.759] vs 0.757 [0.754-0.760]). The category-free NRI was statistically significant (0.089 [0.079-0.098]) with the addition of osteosarcopenia to the CHARGE-AF score.

**Table 3:** Additive Predictive Value of Osteosarcopenia on Atrial Fibrillation Prediction

	AUROC (95% CI)	*P* Value	Harrell's C-index (95% CI)	*P* Value	Category-Free NRI (95% CI)	*P* Value
Model 1	0.758 (0.755-0.760)	-	0.756 (0.754-0.759)	<0.001	-	-
Model 2 (vs Model 1)	0.758 (0.755-0.761)	<0.001	0.757 (0.754-0.760)	<0.001	0.089 (0.079-0.098)	<0.001

Model 1: CHARGE-AF (Cohorts for Heart and Aging Research in Genomic Epidemiology Atrial Fibrillation) score (including age, height, weight, systolic blood pressure, diastolic blood pressure, current smoking, hypertension on medication, diabetes mellitus, heart failure, myocardial infarction).

Model 2: Model 1 + osteosarcopenia.

AUROC = area under the receiver operating characteristic curve; NRI = net reclassification improvement.

In the replication cohort (Supplemental Table 7), adding osteosarcopenia to the model including individual components of CHARGE-AF score did not change the AUROC (0.814-0.815; *P* = 0.414), but significantly improved risk reclassification with a category-free NRI of 16.3% (95% CI: 7.62-24.90%; *P* < 0.001).

### Subgroup analysis

Subgroup analysis, presented in Supplemental Table 8, revealed that the risk of incident AF was significantly associated with certain subgroups. Participants younger than 65 years or BMI less than 30 showed a significant association between osteoporosis and osteosarcopenia and an increased risk of incident AF. In addition, participants with dyslipidemia exhibited a significant association with AF risk compared with those without dyslipidemia (aHR: 1.09; 95% CI: 1.01-1.18 in sarcopenia; aHR: 1.29; 95% CI: 1.04-1.61 in osteoporosis; aHR: 1.47; 95% CI: 1.09-1.99 in osteosarcopenia). In addition, subgroup analysis according to osteoporosis medication status (Supplemental Table 9) showed that osteosarcopenia was significantly associated with incident AF among patients not receiving osteoporosis medication (aHR: 1.40; 95% CI: 1.06-1.85; *P* = 0.018). The comparisons of incident AF risk between the UK Biobank and replication cohort are shown in Supplemental Figure 1. Additional adjustment for socioeconomic factors and detailed alcohol consumption did not materially alter the association between osteosarcopenia and incident AF (Supplemental Table 10). Missingness of key variables and baseline characteristics of included versus excluded participants are summarized in Supplemental Tables 11 and 12, respectively.

## Discussion

The present study offers a novel contribution by evaluating the association of osteosarcopenia with the incidence of AF (Central Illustration). The major findings of this study are as follows: 1) osteoporosis was independently associated with incident AF, whereas sarcopenia alone was not significant after full adjustment; 2) osteosarcopenia was significantly associated with 1.3-fold increased risk of incident AF; 3) the analysis in the replication cohort showed consistent results with the main findings; and 4) osteosarcopenia provided incremental prognostic information beyond the CHARGE-AF score, primarily through improved risk reclassification despite limited improvement in discrimination.

**Central Illustration:**
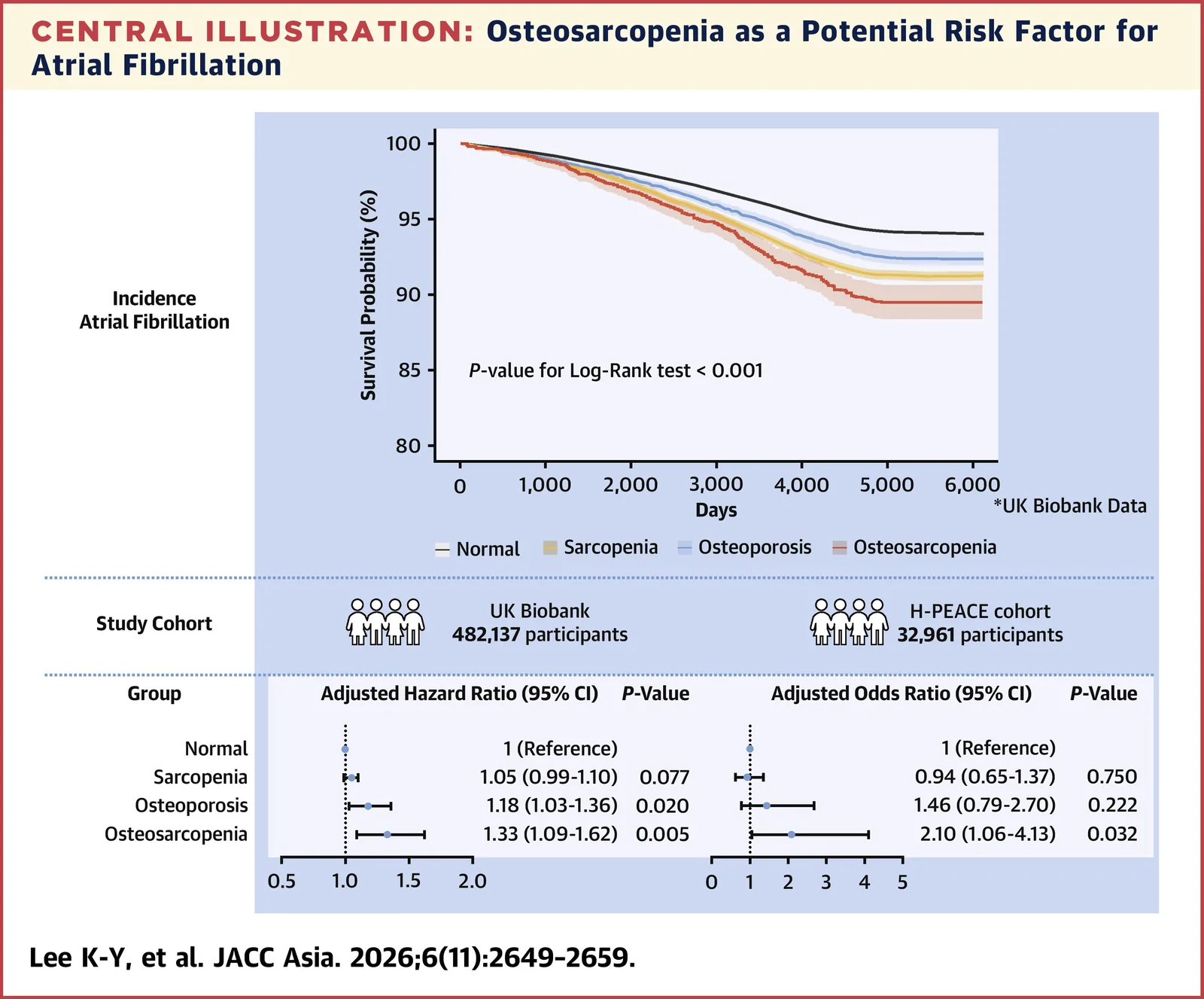
Osteosarcopenia as a Potential Risk Factor for Atrial Fibrillation This central illustration summarizes the association between osteosarcopenia and incident atrial fibrillation in a large population-based cohort. Participants were categorized into reference, sarcopenia, osteoporosis, and osteosarcopenia groups. Osteosarcopenia was independently associated with an increased risk of incident atrial fibrillation compared with the reference group. Sensitivity analyses and external replication confirmed the robustness of the findings. Although discrimination improved only modestly, osteosarcopenia improved risk reclassification beyond traditional risk factors, indicating complementary prognostic value. These findings highlight osteosarcopenia as a geriatric musculoskeletal phenotype associated with elevated atrial fibrillation risk. H-PEACE = Health and Prevention Enhancement.

Our study is the first to report a clear association between osteosarcopenia and incident AF, showing that osteosarcopenia is significantly associated with an increased risk of AF. In our study, the prevalence of osteosarcopenia was only 0.65%, which is considerably lower compared with other studies. Globally, the prevalence of osteosarcopenia ranges from approximately 5% to 37%.^1^ According to a meta-analysis that included 63,369 subjects, the prevalence ranged from 1.5% to 64.3%, depending on sample characteristics such as age and sex, the country where the study was conducted, and the definition of osteosarcopenia used.^17^ Studies with prospective cohort designs, those conducted in Europe, and studies with larger sample sizes tended to report lower prevalence rates. Because our study shares these characteristics, the lower prevalence we observed is consistent with these trends.^17^

Furthermore, osteosarcopenia is more prevalent in older populations, particularly those older than 65 years.^2^^,^^17^^,^^18^ The UK Biobank cohort includes relatively younger participants (mean age of our study population was 56.4 ± 8.1 years), which may partly explain our lower prevalence. In addition, studies that include osteopenia within the definition of osteosarcopenia report higher prevalence rates.^17^ In contrast, we used a more stringent definition, focusing only on patients diagnosed with osteoporosis, which likely contributed to the lower prevalence observed in our study.

There are limited studies evaluating the correlation between osteosarcopenia and CVD, and none have explored the association between osteosarcopenia and AF. Only 1 study has assessed the relationship between osteosarcopenia and coronary artery disease, demonstrating a significant association with coronary artery calcification (aHR: 1.44; 95% CI: 1.061-1.944). This study concluded that osteosarcopenia is linked to an increased risk of incident subclinical coronary atherosclerosis.

Although few studies have examined the relationship between sarcopenia and AF, 1 study reported that sarcopenic patients with overweight or obesity had a higher risk of AF (OR: 4.55; 95% CI: 1.44-14.42).^19^ The association between osteoporosis and AF has been attributed mainly to the potential impact of bisphosphonate use on AF incidence. However, the link between bisphosphonate use and AF remains controversial.^20^, ^21^, ^22^ In addition, our previous study demonstrated that underweight was an independent risk factor for incident AF, even after adjusting for confounders such as chronic lung disease and malignancy.^23^ This supports the notion that low body mass, potentially reflecting reduced muscle mass, may predispose individuals to AF.

We also assessed whether osteosarcopenia provides additional prognostic information beyond established clinical risk factors. Although adding osteosarcopenia to the CHARGE-AF score did not materially improve discrimination as measured by AUROC, it was associated with improved risk reclassification (category-free NRI: 0.089). These findings suggest that osteosarcopenia may offer complementary information for identifying vulnerable individuals at higher risk of AF, even if its incremental contribution to traditional prediction models appears modest. Although the magnitude of association was modest, HRs in this range may still be clinically meaningful in large population-based cohorts, particularly for multifactorial conditions such as AF.

Bone and muscle are closely connected and form the musculoskeletal system, functioning together through reciprocal signaling.^24^ Biochemically, they both originate from mesenchymal stem cells. Hormonal regulation, particularly the growth hormone and insulin-like growth factor 1 axis, plays a crucial role in maintaining bone and muscle mass. The secretion of these hormones declines with aging, leading to reductions in muscle and bone mass and increases in fat mass.^25^^,^^26^ Sarcopenia has also been associated with mitochondrial dysfunction in muscles, cardiomyocyte inflammation, and metabolic syndrome.^27^, ^28^, ^29^ Loss of muscle mass also may influence atrial remodeling via myokine signaling. Myostatin, a myokine involved in the regulation of skeletal muscle growth, has been implicated as a contributor to arrhythmogenic remodeling. Animal models have shown that altered myostatin expression can facilitate a substrate for AF development.^30^ Given this background, it can be hypothesized that osteosarcopenia may contribute to the incidence of AF through atrial inflammation, atrial dysfunction, and metabolic syndrome, which may serve as risk factors for AF.

This study is the first report clearly demonstrating the association between osteosarcopenia and the incidence of AF. In both the UK Biobank and replication cohorts, osteosarcopenia was significantly associated with an increased risk of incident AF (aHR: 1.33; 95% CI: 1.09-1.62; *P* = 0.005 in UK Biobank and adjusted OR: 2.10; 95% CI: 1.06-4.13; *P* = 0.032 in the replication cohort). These consistent findings support the robustness of our primary results and highlight the importance of recognizing osteosarcopenia as a potential risk factor for AF.

Given that this study demonstrated an association between osteosarcopenia and the incidence of AF, further research is warranted to determine whether treating osteosarcopenia could help prevent AF. With the global aging population on the rise, the prevalence of both osteosarcopenia and AF is increasing.^2^^,^^31^^,^^32^ Given that each of these conditions significantly contributes to the rise in medical costs,^33^^,^^34^ it is important to prevent the development and progression of osteosarcopenia.

### Study limitations

First, osteoporosis was defined based on a documented diagnosis before the baseline assessment; therefore, quantitative measurements of bone density obtained via dual-energy X-ray absorptiometry, BIA, computed tomography, or magnetic resonance imaging were not considered. As a result, undiagnosed osteoporosis cases may have been underascertained and misclassified into the reference group, which could have attenuated the observed associations toward the null. Second, AF events were identified using ICD-10 diagnostic codes, without systematic electrocardiogram screening. Given that a substantial proportion of AF is asymptomatic or paroxysmal, incident AF may have been underascertained, raising the possibility of surveillance bias. Such underdetection would likely attenuate the observed association, suggesting that our findings may represent conservative estimates. In addition, because detailed information on health care utilization was not uniformly available, differential AF detection by comorbidity burden cannot be excluded. Third, residual or unmeasured confounding related to the medical conditions, including rheumatologic diseases, steroid use, or hormone replacement therapy, cannot be completely excluded. Fourth, because of its observational design, causality could not be established. Fifth, despite adjusting for several covariates, unmeasured confounders—such as socioeconomic and genetic factors—may still have influenced our findings. Sixth, missing data were handled using complete case analysis. Although some covariates had moderate missingness and the missing completely at random assumption was not fully satisfied, missingness was low for key exposure variables, and excluded participants had a higher comorbidity burden, suggesting that our findings may represent conservative estimates. In addition, comorbidities were represented with limited granularity, which may introduce residual confounding.

## Conclusions

Osteosarcopenia was significantly associated with an increased risk of incident AF and provided incremental prognostic information beyond the CHARGE-AF score, although the gain in discrimination was limited. These findings emphasize the need for tailored AF screening in patients with osteosarcopenia to facilitate earlier diagnosis and treatment.

## Funding Support and Author Disclosures

This research was supported by a grant from the Patient-Centered Clinical Research Coordinating Center (PACEN) funded by the Ministry of Health & Welfare, Republic of Korea (grant number: RS-2021-KH119931), by National Research Foundation of Korea (NRF) grant funded by the Korea government (MSIT) (IRIS RS-2024-00340590), and by the Yuhan Corporation (IIT 076). Dr Choi has received research grants or speaking fees from Abbott, Bayer, BMS/Pfizer, Biosense Webster, Chong Kun Dang, Daewoong Pharmaceutical Co, Daiichi-Sankyo, DeepQure, Dreamtech Co, Ltd, Jeil Pharmaceutical Co Ltd, Medtronic, Samjinpharm, Samsung Electronics Co, Ltd, Seers Technology, Skylabs, and Yuhan Corporation. Dr Lip has been a consultant and speaker for BMS/Pfizer, Boehringer Ingelheim, Anthos, and Daiichi-Sankyo, but no fees are received personally. All other authors have reported that they have no relationships relevant to the contents of this paper to disclose.
