# Supplementary material for: Osteosarcopenia as a Risk Factor for Atrial Fibrillation: A Multi-Cohort Study
Source: JACC Asia. 2026 Sep 16;6(11):2649–59. doi: 10.1016/j.jacasi.2026.08.006 (PMC13750890; doi:10.1016/j.jacasi.2026.08.006)
Supplement: Supplementary file 1 [file mmc1.docx]

**Supplementary Appendix**

**Osteosarcopenia as a Potential Risk Factor for Atrial Fibrillation: A Multi-cohort Study**

**Table of Contents**

1. **Supplemental Tables**

**Supplemental Table 1. Definitions of covariates and outcomes**

**Supplemental Table 2. Incidence and risk of atrial fibrillation according to sarcopenia and osteoporosis**

**Supplemental Table 3. Baseline characteristics in replication cohort**

**Supplemental Table 4. Incidence and risk of atrial fibrillation according to sarcopenia and osteoporosis in replication cohort**

**Supplemental Table 5. Incidence and risk of atrial fibrillation according to groups divided by sarcopenia and osteoporosis status in replication cohort**

**Supplemental Table 6. Logistic regression analysis for risk factors of incident atrial fibrillation in UK Biobank cohort**

**Supplemental Table 7. Additive discriminant ability of osteosarcopenia on atrial fibrillation prediction in replication cohort**

**Supplemental Table 8. Subgroup analysis for risk of atrial fibrillation**

**Supplemental Table 9. Subgroup analysis according to the osteoporosis medication**

**Supplemental Table 10. Association between osteosarcopenia and incident AF after additional adjustment for socioeconomic factors, alcohol amount and alcohol frequency**

**Supplemental Table 11. Missingness of key variables used in the analysis**

**Supplemental Table 12. The comparison of baseline characteristics between included and excluded participants**

2. **Supplemental Figure**

**Supplemental Figure 1. The comparisons of incident AF risk between the UKBB and replication cohort**

**Supplemental Table 1. Definitions of covariates and outcomes**

| **Covariates** | **UK Biobank Field ID** | **Code** |
| --- | --- | --- |
| Age | 21022 |  |
| Sex | 31 |  |
| Body mass index | 21001 |  |
| Standing height | 50 |  |
| Leg_fatfree_right | 23113 |  |
| Leg_fatfree_left | 23117 |  |
| Arm_fatfree_right | 23121 |  |
| Arm_fatfree_left | 23125 |  |
| Handgrip_left | 46 |  |
| Handgrip_right | 47 |  |
| Walking_pace | 924 |  |
| Falls | 2296 |  |
| Current smoking | 20116 |  |
| Townsend deprivation index | 189 |  |
| Income level | 738 |  |
| Indices of multiple deprivation | 26426, 26427 |  |
| Enrollment center | 54 |  |
| Alcohol intake | 1558 |  |
| Physical activity | 22035 |  |
| Diabetes mellitus | 2443, 6153, 6177, 20002, 130708, 130710, 130712, 130714, 41270, 41280 | E11-E14 |
| Hypertension | 6153, 6177, 20002, 2966, 131286, 131288, 131290, 131292, 131294, 41270, 41280 | I10-I13, I15 |
| Dyslipidemia | 6153, 6177, 20002, 130814, 41270, 41280 | E78 |
| Previous MI | 20002, 41270, 41280, 131298, 131300, 131302, 42000 | 1075, I21-I23 |
| Previous HF | 20002, 41270, 41280, 131354 | 1076, I50, I110, I130, I132 |
| Previous stroke | 20002, 6150, 41270, 41280, 131360, 131362, 131364, 131366, 131368, 42006 | 1081, I60-I64 |
| Ca/Vitamin D | 1140852948, 1140870954, 1140909878, 1140871052, 1140877630, 1141146606, 1141180936 |  |
| osteoporosis medication | ## Bisphosphonate (1140868770, 1140868784, 1140868794, 1140882980, 1140910468, 1140910726, 1140910728, 1140913322, 1140922174, 1140927054, 1141175684, 1141176570, 1141180314, 1141173814) ## Teriparatide (1141188794) ## Calcitonin (1140857760, 1140868848) ## Raloxifene (1141168574) ## Estrogen (1140868406, 1140884622, 1141190580) |  |
| **Outcomes** | **UK Biobank Field ID** | **Code** |
| Atrial fibrillation | 20002, 41270, 41280, 131350 | 1471, I48 |

**Supplemental Table 2. Incidence and risk of atrial fibrillation according to sarcopenia and osteoporosis**

|  | **N** | **Event** | **IR** | **Unadjusted HR** | **p-value** | **Adjusted HR (M1)** | **P-value** | **Adjusted HR (M2)** | **P-value** |
| --- | --- | --- | --- | --- | --- | --- | --- | --- | --- |
|  |  |  | **per 1000 PY**  **(95% CI)** | **(95% CI)** |  | **(95% CI)** |  | **(95% CI)** |  |
| **Sarcopenia** |  |  |  |  |  |  |  |  |  |
| Non-sarcopenic | 439,706 | 24,983 | 4.30 (4.25–4.35) | 1 (Reference) | - | 1 (Reference) | - | 1 (Reference) | - |
| Sarcopenia | 42,431 | 3,529 | 6.60 (6.38–6.82) | 1.53 (1.47 - 1.58) | <0.001 | 1.24 (1.19 - 1.28) | <0.001 | 1.05 (1.00 - 1.11) | 0.015 |
| **Osteoporosis** |  |  |  |  |  |  |  |  |  |
| Non-osteoporotic | 464,999 | 27,210 | 4.44 (4.39–4.50) | 1 (Reference) | - | 1 (Reference) | - | 1 (Reference) | - |
| Osteoporosis | 17,138 | 1,302 | 5.95 (5.64–6.29) | 1.34 (1.27 - 1.42) | <0.001 | 1.19 (1.13 - 1.26) | <0.001 | 1.20 (1.05 - 1.37) | 0.008 |

The incidence rate was calculated based on the Kaplan-Meier estimate until median 13.8 years of follow-up.

^*^Adjusted HR was calculated in the multivariable Cox regression model.

Model 1: Adjusted for age and sex.

Model 2: Adjusted for age, sex, BMI, waist circumference, hip circumference, self-reported walking speed, falls in the last year, physical activity, smoking status, alcohol consumption, CCI score, ethnicity, history of hypertension, diabetes mellitus, dyslipidemia, myocardial infarction, heart failure, stroke, levels of hemoglobin, glucose, HbA1c, cholesterol, HDL, LDL, triglycerides, creatinine, albumin, vitamin D, and osteoporosis medication.

Abbreviation: CI, confidence interval; HR, hazard ratio; IR, incidence rate; PY, person-year.

**Supplemental Table 3. Baseline characteristics in the replication cohort**

|  | **Total** | **Reference** | **Sarcopenia** | **Osteoporosis** | **Osteosarcopenia** | **p-value** |
| --- | --- | --- | --- | --- | --- | --- |
|  | N=32,961 | N=26,205 | N=3,734 | N=2,342 | N=680 |  |
| **Age, years** | 54.4 ± 10.7 | 53.8 ± 10.4 | 54.6 ± 12.0 | 58.7 ± 10.0 | 61.0 ± 10.7 | <0.001 |
| **Male sex, n (%)** | 9,211 (27.9%) | 7,968 (30.4%) | 808 (21.6%) | 343 (14.6%) | 92 (13.5%) | <0.001 |
| **Body mass index, kg/m^2^** | 22.5 ± 3.1 | 23.0 ± 3.0 | 19.7 ± 2.1 | 22.6 ± 2.7 | 19.6 ± 2.3 | <0.001 |
| **Waist circumference, cm** | 82.1 ± 8.7 | 83.1 ± 8.6 | 75.8 ± 7.3 | 82.4 ± 7.8 | 75.8 ± 7.3 | <0.001 |
| **Current smoker, n (%)** | 4,101 (14.6%) | 3,552 (15.8%) | 369 (11.6%) | 133 (7.2%) | 47 (8.3%) | <0.001 |
| **Daily drinking, n (%)** | 1,003 (3.7%) | 843 (3.9%) | 120 (4.0%) | 29 (1.7%) | 11 (2.2%) | <0.001 |
| **Hypertension, n (%)** | 6,117 (22.9%) | 5,003 (23.6%) | 485 (16.2%) | 503 (26.0%) | 126 (22.0%) | <0.001 |
| **Diabetes mellitus, n (%)** | 2,156 (8.1%) | 1,687 (7.9%) | 255 (8.5%) | 171 (8.8%) | 43 (7.5%) | 0.39 |
| **Dyslipidemia, n (%)** | 6,617 (24.7%) | 5,195 (24.5%) | 622 (20.7%) | 628 (32.5%) | 172 (30.0%) | <0.001 |
| **Systolic blood pressure, mmHg** | 116.6 ± 14.8 | 116.8 ± 14.6 | 114.4 ± 15.3 | 118.1 ± 15.1 | 118.4 ± 16.6 | <0.001 |
| **Diastolic blood pressure, mmHg** | 74.3 ± 10.0 | 74.7 ± 10.0 | 72.3 ± 9.8 | 74.2 ± 9.7 | 72.6 ± 10.2 | <0.001 |
| **Hemoglobin concentration, g/dL** | 13.8 ± 1.4 | 13.8 ± 1.4 | 13.7 ± 1.3 | 13.6 ± 1.1 | 13.5 ± 1.1 | <0.001 |
| **Fasting glucose, mg/dL** | 98.1 ± 17.7 | 98.5 ± 17.6 | 96.0 ± 19.1 | 97.1 ± 15.2 | 96.8 ± 21.0 | <0.001 |
| **HbA1c, %** | 5.7 ± 0.6 | 5.7 ± 0.6 | 5.6 ± 0.6 | 5.7 ± 0.5 | 5.7 ± 0.6 | <0.001 |
| **Total cholesterol, mg/dL** | 195.8 ± 36.9 | 195.0 ± 36.8 | 199.6 ± 37.6 | 197.5 ± 35.7 | 201.4 ± 38.8 | <0.001 |
| **HDL, mg/dL** | 57.6 ± 13.1 | 56.8 ± 12.9 | 61.7 ± 13.6 | 58.3 ± 12.7 | 63.6 ± 13.3 | <0.001 |
| **LDL, mg/dL** | 95.6 ± 58.5 | 97.7 ± 60.4 | 84.1 ± 45.8 | 94.7 ± 56.5 | 82.6 ± 40.5 | <0.001 |
| **TG, mg/dL** | 120.2 ± 32.4 | 120.2 ± 32.4 | 120.3 ± 32.4 | 120.4 ± 31.5 | 119.6 ± 33.9 | 0.96 |
| **Creatinine, mg/dL** | 0.8 ± 0.2 | 0.8 ± 0.2 | 0.7 ± 0.2 | 0.7 ± 0.2 | 0.7 ± 0.2 | <0.001 |
| **Albumin, g/dL** | 4.4 ± 0.3 | 4.4 ± 0.3 | 4.5 ± 0.3 | 4.4 ± 0.3 | 4.4 ± 0.3 | <0.001 |
| **Osteoporosis medication, n (%)** | 2,205 (8.2%) | 0 (0.0%) | 0 (0.0%) | 1,817 (80.9%) | 388 (61.9%) | <0.001 |

Data summarized as: Mean ± SD or %

Abbreviations are as in Table 1.

**Supplemental Table 4. Incidence and risk of atrial fibrillation according to sarcopenia and osteoporosis in replication cohort**

|  | **N** | **Event** | **Rate, %** | **Unadjusted OR** | **P-value** | **Adjusted OR (M1)** | **P-value** |
| --- | --- | --- | --- | --- | --- | --- | --- |
|  |  |  |  | **(95% CI)** |  | **(95% CI)** |  |
| **Sarcopenia** |  |  |  |  |  |  |  |
| Non-sarcopenic | 28,547 | 585 | 2.05 | 1 (Reference) | - | 1 (Reference) | - |
| Sarcopenia | 4,414 | 87 | 1.97 | 0.96 (0.77-1.21) | 0.732 | 1.05 (0.76 - 1.46) | 0.771 |
| **Osteoporosis** |  |  |  |  |  |  |  |
| Non-osteoporotic | 29,939 | 598 | 2.00 | 1 (Reference) | - | 1 (Reference) | - |
| Osteoporosis | 3,022 | 74 | 2.45 | 1.23 (0.97-1.57) | 0.095 | 1.70 (0.99-2.92) | 0.055 |

Adjusted ORs were calculated in the multivariable logistic regression model.

Model 1: Adjusted for age, sex, BMI, waist circumference, smoking status, alcohol consumption, systolic blood pressure, diastolic blood pressure, levels of hemoglobin, glucose, HbA1c, total cholesterol, triglycerides, creatinine, albumin, and osteoporosis medication.

Abbreviations: OR, odds ratio; otherwise as in Table 2.

**Supplemental Table 5. Incidence and risk of atrial fibrillation according to groups divided by sarcopenia and osteoporosis status in replication cohort**

|  | **N** | **Event** | **Rate, %** | **Unadjusted OR** | | **Adjusted OR (M1)** | |
| --- | --- | --- | --- | --- | --- | --- | --- |
|  |  |  |  | **OR (95% CI)** | **p-value** | **OR (95% CI)** | **p-value** |
| **Reference** | 26,205 | 532 | 2.03 | 1 (Reference) |  | 1 (Reference) |  |
| **Sarcopenia** | 3,734 | 66 | 1.77 | 0.87 (0.67-1.12) | 0.284 | 0.94 (0.65-1.37) | 0.750 |
| **Osteoporosis** | 2,342 | 53 | 2.26 | 1.12 (0.84-1.49) | 0.446 | 1.46 (0.79-2.70) | 0.222 |
| **Osteosarcopenia** | 680 | 21 | 3.09 | 1.54 (0.99-2.40) | 0.057 | 2.10 (1.06-4.13) | 0.032 |

Adjusted ORs were calculated in the multivariable logistic regression model.

Model 1: Adjusted for age, sex, BMI, waist circumference, smoking status, alcohol consumption, systolic blood pressure, diastolic blood pressure, levels of hemoglobin, glucose, HbA1c, total cholesterol, triglycerides, creatinine, albumin, and osteoporosis medication.

Abbreviations: OR, odds ratio; otherwise as in Table 2.

**Supplemental Table 6. Logistic regression analysis for risk factors of incident atrial fibrillation in UK Biobank cohort**

|  | **Univariable** | | **Multivariable** | |
| --- | --- | --- | --- | --- |
|  | **OR (95% CI)** | **P-value** | **OR (95% CI)** | **p-value** |
| **Sarcopenia** | 1.50 (1.44 - 1.56) | <0.001 | 1.11 (1.06 - 1.17) | <0.001 |
| **Osteoporosis** | 1.29 (1.21 - 1.38) | <0.001 | 1.22 (1.13 - 1.32) | <0.001 |
| **Osteosarcopenia** | 1.78 (1.58 - 2.00) | <0.001 | 1.29 (1.11 - 1.50) | 0.001 |
| **Age, year** | 1.12 (1.12 - 1.12) | <0.001 | 1.11 (1.10 - 1.11) | <0.001 |
| **Sex, male** | 2.01 (1.96 - 2.06) | <0.001 | 1.91 (1.85 - 1.96) | <0.001 |
| **Obesity, BMI ≥30** | 1.80 (1.76 - 1.85) | <0.001 | 1.58 (1.53 - 1.63) | <0.001 |
| **Hypertension** | 2.69 (2.63 - 2.76) | <0.001 | 1.57 (1.52 - 1.62) | <0.001 |
| **Diabetes** | 2.28 (2.19 - 2.37) | <0.001 | 1.11 (1.06 - 1.17) | <0.001 |
| **Dyslipidemia** | 2.51 (2.44 - 2.57) | <0.001 | 1.10 (1.06 - 1.14) | <0.001 |
| **Previous history of HF** | 4.96 (4.41 - 5.58) | <0.001 | 2.20 (1.91 - 2.53) | <0.001 |
| **Daily drinking** | 1.28 (1.24 - 1.32) | <0.001 | 1.08 (1.05 - 1.12) | <0.001 |
| **Smoking status** | 1.06 (1.02 - 1.11) | 0.001 | 1.26 (1.20 - 1.31) | <0.001 |
| **Physical activity** | 1.02 (0.99 - 1.05) | 0.174 | 1.02 (0.99 - 1.05) | 0.112 |

Abbreviations: BMI, body mass index; HF, heart failure; OR, odds ratio.

**Supplemental Table 7. Additive discriminant ability of osteosarcopenia on atrial fibrillation prediction in replication cohort**

|  | **AUROC** | **P-value** | **Category-free NRI** | **P-value** |
| --- | --- | --- | --- | --- |
|  | **(95% CI)** |  | **(95% CI)** |  |
| **Model 1** | 0.814 (0.796-0.832) | - | - | - |
| **Model 2 (vs. Model 1)** | 0.815 (0.797-0.832) | 0.414 | 16.3% (7.62%-24.90%) | <0.001 |

Model 1: Components of CHARGE-AF score (age, height, weight, SBP, DBP, current smoking, HTN on med, DM, and MI)

Model 2: Model 1 + osteosarcopenia

Abbreviations: AF, atrial fibrillation; AUROC, Area Under ROC Curve; DBP, diastolic blood pressure; DM, diabetes mellitus; HTN, hypertension; MI, myocardial infarction; NRI, Net reclassification improvement; ROC, Receiver Operating Characteristic; SBP, systolic blood pressure.

**Supplemental Table 8. Subgroup analysis for risk of atrial fibrillation**

| **Subgroup** |  | **groups** | **Case** | **Event** | **IR, per 1000 PY** | **HR (95% CI)** | **p-value** | ***p for interaction*** |
| --- | --- | --- | --- | --- | --- | --- | --- | --- |
| **Age** | <65 years | Reference | 353299 | 14562 | 3.07 | Reference |  | <0.001 |
|  |  | Sarcopenia | 27604 | 1619 | 4.54 | 1.04 (0.96 - 1.12) | 0.327 |  |
|  |  | Osteoporosis | 9659 | 525 | 4.15 | 1.24 (1.02 - 1.51) | 0.029 |  |
|  |  | Osteosarcopenia | 1809 | 135 | 5.99 | 1.38 (1.03 - 1.85) | 0.031 |  |
|  | ≥65 years | Reference | 72415 | 9421 | 10.6 | Reference |  |  |
|  |  | Sarcopenia | 11681 | 1608 | 11.5 | 1.04 (0.97 - 1.12) | 0.250 |  |
|  |  | Osteoporosis | 4333 | 475 | 8.85 | 1.07 (0.88 - 1.31) | 0.492 |  |
|  |  | Osteosarcopenia | 1337 | 167 | 10.5 | 1.22 (0.93 - 1.59) | 0.148 |  |
| **Sex** | Male | Reference | 200486 | 15508 | 5.96 | Reference |  | 0.001 |
|  |  | Sarcopenia | 14578 | 1642 | 9.31 | 1.04 (0.97 - 1.11) | 0.276 |  |
|  |  | Osteoporosis | 2284 | 292 | 10.7 | 1.19 (0.94 - 1.50) | 0.141 |  |
|  |  | Osteosarcopenia | 436 | 63 | 14 | 1.27 (0.86 - 1.89) | 0.227 |  |
|  | Female | Reference | 225228 | 8475 | 2.8 | Reference |  |  |
|  |  | Sarcopenia | 24707 | 1585 | 4.95 | 1.04 (0.96 - 1.13) | 0.313 |  |
|  |  | Osteoporosis | 11708 | 708 | 4.63 | 1.13 (0.94 - 1.35) | 0.184 |  |
|  |  | Osteosarcopenia | 2710 | 239 | 7.05 | 1.24 (0.99 - 1.56) | 0.062 |  |
| **Obesity** | BMI <30 | Reference | 323933 | 15540 | 3.61 | Reference |  | 0.584 |
|  |  | Sarcopenia | 27379 | 1879 | 5.39 | 1.02 (0.95 - 1.09) | 0.607 |  |
|  |  | Osteoporosis | 11638 | 744 | 4.93 | 1.18 (1.00 - 1.39) | 0.046 |  |
|  |  | Osteosarcopenia | 2430 | 197 | 6.55 | 1.27 (1.01 - 1.62) | 0.045 |  |
|  | BMI ≥30 | Reference | 101781 | 8443 | 6.39 | Reference |  |  |
|  |  | Sarcopenia | 11906 | 1348 | 9.11 | 1.11 (1.02 - 1.20) | 0.016 |  |
|  |  | Osteoporosis | 2354 | 256 | 8.72 | 1.12 (0.86 - 1.47) | 0.404 |  |
|  |  | Osteosarcopenia | 716 | 105 | 12.6 | 1.43 (1.01 - 2.02) | 0.047 |  |
| **Hypertension** | No | Reference | 305681 | 12044 | 2.94 | Reference |  | 0.010 |
|  |  | Sarcopenia | 24029 | 1301 | 4.18 | 1.02 (0.95 - 1.11) | 0.568 |  |
|  |  | Osteoporosis | 9906 | 533 | 4.1 | 1.15 (0.94 - 1.40) | 0.182 |  |
|  |  | Osteosarcopenia | 1905 | 125 | 5.18 | 1.05 (0.77 - 1.43) | 0.738 |  |
|  | Yes | Reference | 120033 | 11939 | 7.79 | Reference |  |  |
|  |  | Sarcopenia | 15256 | 1926 | 10.4 | 1.06 (0.99 - 1.13) | 0.099 |  |
|  |  | Osteoporosis | 4086 | 467 | 9.27 | 1.20 (0.99 - 1.46) | 0.070 |  |
|  |  | Osteosarcopenia | 1241 | 177 | 12.4 | 1.56 (1.21 - 2.01) | 0.001 |  |
| **Diabetes mellitus** | No | Reference | 405212 | 21650 | 4.03 | Reference |  | 0.001 |
|  |  | Sarcopenia | 35157 | 2646 | 5.9 | 1.03 (0.98 - 1.09) | 0.251 |  |
|  |  | Osteoporosis | 13411 | 924 | 5.33 | 1.13 (0.98 - 1.32) | 0.095 |  |
|  |  | Osteosarcopenia | 2879 | 269 | 7.61 | 1.33 (1.08 - 1.64) | 0.007 |  |
|  | Yes | Reference | 20502 | 2333 | 9.18 | Reference |  |  |
|  |  | Sarcopenia | 4128 | 581 | 12.1 | 1.13 (0.99 - 1.29) | 0.061 |  |
|  |  | Osteoporosis | 581 | 76 | 11.1 | 1.68 (1.10 - 2.58) | 0.017 |  |
|  |  | Osteosarcopenia | 267 | 33 | 10.8 | 1.33 (0.73 - 2.41) | 0.350 |  |
| **Dyslipidemia** | No | Reference | 348657 | 15757 | 3.38 | Reference |  | <0.001 |
|  |  | Sarcopenia | 28073 | 1760 | 4.87 | 1.00 (0.93 - 1.07) | 0.963 |  |
|  |  | Osteoporosis | 11040 | 668 | 4.64 | 1.10 (0.92 - 1.32) | 0.309 |  |
|  |  | Osteosarcopenia | 2190 | 169 | 6.2 | 1.20 (0.92 - 1.55) | 0.171 |  |
|  | Yes | Reference | 77057 | 8226 | 8.46 | Reference |  |  |
|  |  | Sarcopenia | 11212 | 1467 | 10.9 | 1.09 (1.01 - 1.18) | 0.021 |  |
|  |  | Osteoporosis | 2952 | 332 | 9.1 | 1.29 (1.04 - 1.61) | 0.023 |  |
|  |  | Osteosarcopenia | 956 | 133 | 12 | 1.47 (1.09 - 1.99) | 0.012 |  |

Data summarized as: Mean ± SD or %

Abbreviations: BMI, body mass index.

**Supplemental Table 9. Subgroup analysis according to the osteoporosis medication**

| **Subgroup** | **groups** | **Case** | **Event** | **IR,  per 1000 PY** | **HR (95% CI)** | **p-value** |
| --- | --- | --- | --- | --- | --- | --- |
| **Medication for osteoporosis** | **Reference** | 425714 | 23983 | 4.26 | 1 (Reference) |  |
|  | **Osteoporosis on med** | 10013 | 677 | 5.24 | 1.11 (1.00 - 1.24) | 0.045 |
|  | **Osteoporosis not on med** | 3979 | 323 | 6.32 | 1.16 (1.00 - 1.35) | 0.051 |
|  | **Osteosarcopenia, on med for osteoporosis** | 2195 | 208 | 7.77 | 1.22 (1.00 - 1.48) | 0.054 |
|  | **Osteosarcopenia, not on med for osteoporosis** | 951 | 94 | 8.10 | 1.40 (1.06 - 1.85) | 0.018 |

Data summarized as: Mean ± SD or %

Abbreviations: BMI, body mass index HR, hazard ratio; IR, incidence rate.

**Supplemental Table 10. Association between osteosarcopenia and incident AF after additional adjustment for socioeconomic factors, alcohol amount and alcohol frequency**

|  | **Adjusted HR (M2)** | | **Adjusted HR (M4)** | | **Adjusted HR (M5)** | |
| --- | --- | --- | --- | --- | --- | --- |
|  | **HR (95% CI)** | **p-value** | **HR (95% CI)** | **p-value** | **HR (95% CI)** | **p-value** |
| **Reference** | 1 (Reference) |  | 1 (Reference) |  | 1 (Reference) |  |
| **Sarcopenia** | 1.05 (0.99 - 1.10) | 0.077 | 1.04 (0.99 - 1.10) | 0.127 | 1.01 (0.95 - 1.08) | 0.668 |
| **Osteoporosis** | 1.18 (1.03 - 1.36) | 0.020 | 1.17 (1.00 - 1.36) | 0.048 | 1.22 (1.02 - 1.45) | 0.027 |
| **Osteosarcopenia** | 1.33 (1.09 - 1.62) | 0.005 | 1.34 (1.08 - 1.66) | 0.009 | 1.44 (1.10 - 1.88) | 0.007 |

Model 2: Adjusted for age, sex, BMI, waist circumference, hip circumference, self-reported walking speed, falls in the last year, physical activity, smoking status, alcohol consumption, CCI score, ethnicity, history of hypertension, diabetes mellitus, dyslipidemia, myocardial infarction, heart failure, stroke, levels of hemoglobin, glucose, HbA1c, cholesterol, HDL, LDL, triglycerides, creatinine, albumin, vitamin D, and osteoporosis medication.

Model 4: Model 2 + Townsend deprivation index and household income before tax.

Model 5: Model 2 + alcohol amount and alcohol frequency.

Abbreviation: CI, confidence interval; HR, hazard ratio; IR, incidence rate; PY, person-year.

**Supplemental Table 11. Missingness of key variables used in the analysis**

|  | **Missing (%)** |
| --- | --- |
| **ALM/height^2^** | 10,620 (2.11) |
| **Hand grip strength** | 4,395 (0.87) |
| **Osteoporosis** | 0 (0) |
| **Age, years** | 3 (0.00) |
| **Male sex, n (%)** | 0 (0) |
| **Body mass index, kg/m^2^** | 3107 (0.62) |
| **Waist circumference, cm** | 2163 (0.43) |
| **Hip circumference, cm** | 2222 (0.44) |
| **Self-reported walk speed** | 4678 (0.93) |
| **Falls in last year** | 2670 (0.53) |
| **Regular physical activity, n (%)** | 100104 (19.93) |
| **Current smoker, n (%)** | 0 (0) |
| **Daily drinking, n (%)** | 0 (0) |
| **Hypertension, n (%)** | 0 (0) |
| **Diabetes mellitus, n (%)** | 0 (0) |
| **Dyslipidemia, n (%)** | 0 (0) |
| **Previous history of MI, n (%)** | 0 (0) |
| **Previous history of HF, n (%)** | 0 (0) |
| **Previous history of stroke, n (%)** | 0 (0) |
| **Hemoglobin concentration, g/dL** | 24327 (4.84) |
| **Fasting glucose, mmol/L** | 72917 (14.51) |
| **HbA1c, mmol/mol** | 35995 (7.16) |
| **Total cholesterol, mmol/L** | 32906 (6.55) |
| **HDL, mmol/L** | 72613 (14.45) |
| **LDL, mmol/L** | 33789 (6.73) |
| **TG, mmol/L** | 33281 (6.62) |
| **Creatinine, µmol/L** | 33138 (6.6) |
| **Albumin, g/L** | 72409 (14.41) |
| **Osteoporosis medication, n (%)** | 0 (0) |

Data are presented as n (%) of missing values among the initial cohort (N = 502,386).

Abbreviations: ALM, appendicular lean mass; otherwise as in Table 1.

**Supplemental Table 12. The comparison of baseline characteristics between included and excluded participants**

|  | **Total** | **Included** | **Excluded** | **p-value** |
| --- | --- | --- | --- | --- |
|  | N= 502386 | N= 482137 | N= 20249 |  |
| **Age, years** | 56.5 ± 8.1 | 56.4 ± 8.1 | 59.2 ± 7.8 | <0.001 |
| **Male sex, n (%)** | 229076 (45.6) | 217784 (45.2) | 11292 (55.8) | <0.001 |
| **Body mass index, kg/m^2^** |  |  |  | <0.001 |
| **< 18.5** | 2626 (0.5) | 2471 (0.5) | 155 (0.9) |  |
| **18.5 ≤ BMI < 25** | 162360 (32.5) | 157827 (32.7) | 4533 (26.4) |  |
| **25 ≤ BMI < 30** | 212070 (42.5) | 205082 (42.5) | 6988 (40.8) |  |
| **≥ 30** | 122223 (24.5) | 116757 (24.2) | 5466 (31.9) |  |
| **Waist circumference, cm** | 90.3 ± 13.5 | 90.1 ± 13.4 | 95.0 ± 15.0 | <0.001 |
| **Hip circumference, cm** | 103.4 ± 9.2 | 103.3 ± 9.2 | 105.4 ± 10.8 | <0.001 |
| **Self-reported walk speed** |  |  |  | <0.001 |
| **Slow pace** | 40897 (8.2) | 36813 (7.7) | 4084 (21.6) |  |
| **Steady average pace** | 262759 (52.8) | 253224 (52.9) | 9535 (50.4) |  |
| **Brisk pace** | 194052 (39.0) | 188756 (39.4) | 5296 (28.0) |  |
| **Falls in last year** |  |  |  | <0.001 |
| **0** | 400648 (80.2) | 386777 (80.6) | 13871 (70.7) |  |
| **1** | 65943 (13.2) | 62786 (13.1) | 3157 (16.1) |  |
| **2+** | 33125 (6.6) | 30523 (6.4) | 2602 (13.3) |  |
| **Current smoker, n (%)** | 52962 (10.5) | 50837 (10.5) | 2125 (10.5) | 0.83 |
| **Daily drinking, n (%)** | 101749 (20.3) | 97715 (20.3) | 4034 (19.9) | 0.235 |
| **Hypertension, n (%)** | 150284 (29.9) | 140616 (29.2) | 9668 (47.7) | <0.001 |
| **Diabetes mellitus, n (%)** | 27722 (5.5) | 25478 (5.3) | 2244 (11.1) | <0.001 |
| **Dyslipidemia, n (%)** | 99730 (19.9) | 92177 (19.1) | 7553 (37.3) | <0.001 |
| **Previous history of MI, n (%)** | 12184 (2.4) | 10306 (2.1) | 1878 (9.3) | <0.001 |
| **Previous history of HF, n (%)** | 2760 (0.5) | 1565 (0.3) | 1195 (5.9) | <0.001 |
| **Previous history of stroke, n (%)** | 9096 (1.8) | 7739 (1.6) | 1357 (6.7) | <0.001 |
| **Hemoglobin concentration, g/dL** | 14.2 ± 1.2 | 14.2 ± 1.2 | 14.2 ± 1.3 | <0.001 |
| **Fasting glucose, mmol/L** | 5.1 ± 1.2 | 5.1 ± 1.2 | 5.3 ± 1.7 | <0.001 |
| **HbA1c, mmol/mol** | 36.1 ± 6.8 | 36.1 ± 6.7 | 37.8 ± 8.8 | <0.001 |
| **Total cholesterol, mmol/L** | 5.7 ± 1.1 | 5.7 ± 1.1 | 5.3 ± 1.2 | <0.001 |
| **HDL, mmol/L** | 1.4 ± 0.4 | 1.5 ± 0.4 | 1.4 ± 0.4 | <0.001 |
| **LDL, mmol/L** | 3.6 ± 0.9 | 3.6 ± 0.9 | 3.3 ± 0.9 | <0.001 |
| **TG, mmol/L** | 1.7 ± 1.0 | 1.7 ± 1.0 | 1.8 ± 1.1 | <0.001 |
| **Creatinine, µmol/L** | 72.3 ± 18.6 | 72.2 ± 18.0 | 76.2 ± 29.0 | <0.001 |
| **Albumin, g/L** | 45.2 ± 2.6 | 45.2 ± 2.6 | 44.6 ± 2.8 | <0.001 |
| **Osteoporosis medication, n (%)** | 12936 (2.6) | 12208 (2.5) | 728 (3.6) | <0.001 |

Data summarized as: Mean ± SD or %

Abbreviations are as in Table 1.

**Supplemental Figure 1. The comparisons of incident AF risk between the UKBB and replication cohort**

Abbreviations: HR, hazard ratio; N, reference group; O, osteoporosis; OR, odds ratio; OS, osteosarcopenia; S, sarcopenia; UKBB, UK Biobank.
